# Understanding the Impact of Approved but Unfunded Vaccine Status on Parental Acceptance of an Adjuvanted Seasonal Influenza Vaccine for Infants: Results from the Pediatric Influenza Vaccination Optimization Trial (PIVOT)–III

**DOI:** 10.3390/vaccines10101769

**Published:** 2022-10-21

**Authors:** William A. Fisher, Vladimir Gilca, Michelle Murti, Alison Orth, Hartley Garfield, Paul Roumeliotis, Emmanouil Rampakakis, Vivien Brown, John Yaremko, Paul Van Buynder, Constantina Boikos, James A. Mansi

**Affiliations:** 1 Department of Obstetrics and Gynaecology, Western University, London, ON N6A 3K7, Canada; 2 Département de Médecine Sociale Et Préventive, Faculté de Médecine, Institut Nationale de Sante Publique du Québec, Université Laval, Québec City, QC G1V 5B3, Canada; 3 Dalla Lana School of Public Health, University of Toronto, Toronto, ON M5T 3M7, Canada; 4 Fraser Health Authority, Vancouver, BC V3T 0H1, Canada; 5 The Hospital for Sick Children, University of Toronto, Toronto, ON M5G 1X8, Canada; 6 Eastern Ontario Health Unit, Cornwall, ON K6J 5T1, Canada; 7 JSS Medical Research, Montreal, QC H4S 1N8, Canada; 8 Department of Family and Community Medicine, University of Toronto, Toronto, ON M5G 1V7, Canada or; 9 The Montreal Children’s Hospital, Montreal, QC H4A 3J1, Canada; 10 Department of Pediatrics, McGill University, Montreal, QC H3A 0G4, Canada; 11 School of Medicine, Griffith University, Perth, WA 6009, Australia; 12 Seqirus, Montreal, QC H9H 4M7, Canada

**Keywords:** approved vaccine, unfunded vaccine, parental acceptance, vaccine hesitancy, influenza

## Abstract

The adjuvanted trivalent influenza vaccine (aTIV) provides enhanced protection against influenza for infants but is not publicly funded (NPF). The objective of this prospective cohort study of parents with children 6 through 23 months of age was to understand how NPF status influences parental perceptions of approved but unfunded vaccines and their intentions to vaccinate. At healthy baby visits, clinicians provided parents with information about influenza and vaccination. Before and after these interactions, a research nurse assessed parents’ intentions to vaccinate their children and their beliefs about the safety, efficacy, and necessity of vaccinating their children with aTIV in both publicly funded (PF) and NPF settings. Overall, 15 community practice clinics (n = 15 physicians) and nine public health clinics (n = 9 nurses) recruited 207 parents. The percentage of parents intending to immunize their children with aTIV decreased from 72% (vaccine PF, free of charge), to 42% (NPF, $25 per dose), to 27% (NPF, $50 per dose). Funding status strongly influenced whether parents perceived immunization with aTIV to be necessary, safe, and effective. Information on influenza and influenza vaccines should be provided to parents routinely to allow for well-informed decisions on the suitability of specific influenza vaccines for their child.

## 1. Introduction

Children < 2 years of age and adults ≥ 65 years with comorbid conditions have the highest rates of influenza-related morbidity and mortality [1,2,3]. Children 6 months through 4 years of age as a group also have an elevated risk of influenza-related complications [4]. As such, Canada’s National Advisory Committee on Immunization (NACI), the UK’s Joint Committee on Vaccines and Immunization, and the US Advisory Committee on Immunization Practices consider children aged 6 months through 5 years a priority for seasonal influenza immunization [5,6]. Nonetheless, many children at high risk of complications from influenza infection do not receive the vaccine [7], and its use in healthy infants is not at optimal levels [8].

The Canadian vaccine approval process, like that in many settings, is tied to increasingly complex vaccine technology, production cost, and competing financial demands in the public health sector. The regulatory approval process for a vaccine generally begins with the national biologics regulator granting market authorization based on a review of the properties and performance of the vaccine [9]. While the vaccine may be used at this point, consideration for a vaccine to be included within a publicly funded (PF) vaccine program requires additional review by the National Immunization Technical Advisory Group (NITAG). The NITAG considers the public health impact of the approved vaccine in considering recommendation for population use. Finally, for an approved vaccine to achieve publicly funded (PF) status in a vaccine program, funding approval from government departments with competing funding demands is required [9]. In Canada, while there is a single NITAG, each of the 13 Canadian provinces and territories is responsible for the funding and scope of its immunization program [9]. Although the entirety of the evidence evaluated by NACI is available to each province and territory, funding decisions differ between the different provincial and territorial ministries of finance, leading to a fragmented tapestry of PF immunization schedules and programs across the country.

Currently, three different types of influenza vaccine are approved for use in Canadian children 6 through 23 months of age and recommended by NACI: trivalent inactivated influenza vaccines (TIV), quadrivalent inactivated influenza vaccines (QIV), and the MF59^®^ adjuvanted trivalent inactivated influenza vaccine (aTIV; Fluad^™^, Seqirus UK Vaccines). In infants and young children, a single dose of aTIV has been shown to induce substantially higher hemagglutination inhibition (HI) titers more rapidly and with longer persistence than nonadjuvanted TIV, with consistently higher seroprotection rates at increased HI titer thresholds against both homologous and heterologous influenza strains [10]. Moreover, the aTIV vaccine, which has been shown to be well tolerated with no pattern of associated serious adverse events, was significantly more effective than TIV in preventing polymerase chain reaction (PCR)-confirmed influenza [11,12]. However, the pediatric formulation of aTIV is one of many vaccines not currently included in PF vaccination programs. This is not unusual, as there is often a delay of several years between approval of a vaccine and its inclusion in the PF vaccine schedule [11].

Offering a choice of pediatric influenza vaccine options to parents allows the parent to balance the out-of-pocket cost of an approved but not publicly funded (NPF) vaccine against the additional protection that it may offer. The primary objective of this study was to explore how PF and NPF statuses influence parental intentions to vaccinate with aTIV and perceptions of this vaccine’s safety, importance, and effectiveness. While aTIV received approval from Canadian regulatory authorities in 2015, this study was conducted before the vaccine was available for clinical practice.

## 2. Materials and Methods

**Figure 1:**
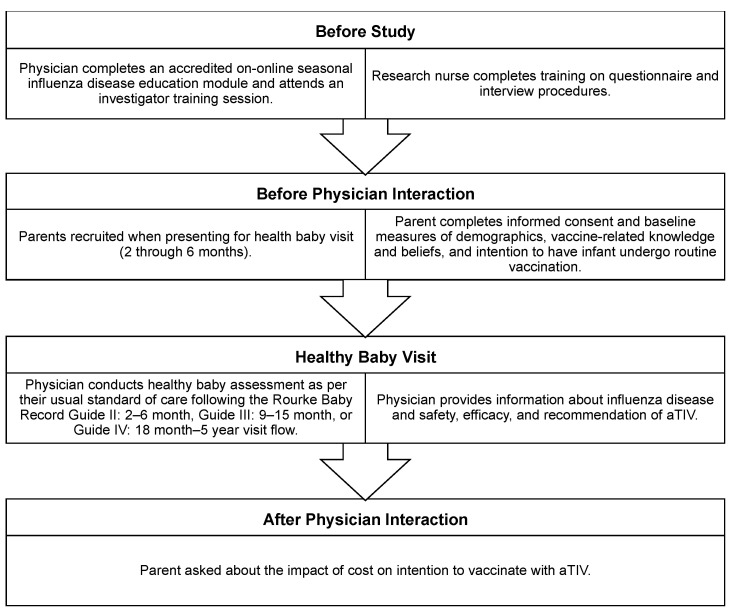
Study recruitment procedures and protocol flow.

The research question was addressed with a prospective cohort survey design conducted during the 2015–2016 influenza season. Briefly, the study population consisted of parents of infants aged 6 through 23 months presenting for a scheduled “healthy baby visit.” Parents were recruited and interviewed by a research nurse before and after a clinician provided information about influenza and aTIV (Figure 1). In addition to being the biological parent or legal caregiver of the infant, survey participants were required to be able and willing to provide written informed consent (in English or French) and to be able and willing to complete two sets of questionnaires before and after the health care provider visit. Parents who had already participated in previous vaccine acceptance studies were excluded from participation to prevent the introduction of bias due to sensitization from prior discussions about aTIV.

The research protocol received ethics approval by the Western University Health Sciences Research Ethics Board, the Fraser Health Research Ethics Board, and IRB Services.

Summary statistics including the mean and standard deviation for continuous variables and frequency distributions for categorical variables were produced for baseline participant sociodemographic characteristics. Similarly, parents’ intention to vaccinate and vaccine-related beliefs were described using frequency distributions. Parents’ intention to vaccinate their infants with aTIV was compared between groups of interest using logistic regression. All analyses were conducted using SPSS version 24 (IBM Corp., Armonk, NY, USA).

## 3. Results

### 3.1. Sample Characteristics

A total of 24 community practice and public health clinics across Canada participated; 15 community practice clinics (15 physicians) enrolled 136 parents whilst 9 public health clinics (9 nurses) enrolled 71 parents (N = 207). Baseline demographics were similar (Table 1).

### 3.2. Parental Acceptance

**Figure 2:**
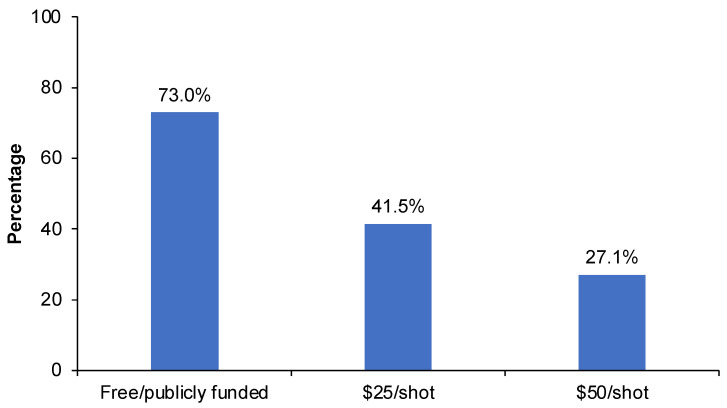
Impact of vaccine cost on parental intention to vaccinate, shown as the percentage of parents responding “strongly agree” or “moderately agree” to the following questions: “I intend to give my baby the aTIV vaccine if it is provided free of charge through public health programs” (free/publicly funded). “I intend to give my baby the aTIV vaccine if the vaccine costs $25 per shot (up to a maximum of $50)” ($25/shot). “I intend to give my baby the aTIV vaccine if the vaccine costs $50 per shot (up to a maximum of $100)” ($50/shot).

PF and NPF status and cost of aTIV vaccination to the parents was a major factor influencing parental intention to vaccinate. The percentage of parents intending to immunize their children with aTIV decreased from 72% if PF (“provided free of charge through public health programs)” to 42% if NPF + $25 (“the vaccine cost $25 per shot (up to a maximum of $50 for 2 shots if your child has not received a flu vaccine before”) to 27% NPF + $50 if “the vaccine cost $50 per shot (up to a maximum of $100 for 2 shots if your child has not received a flu vaccine before)” (Figure 2).

Univariate logistic regression analysis indicated that family annual income was not related to parental acceptance of NPF vaccination at the $25 or $50 per dose level (for a total of $50 or $100 for two doses, respectively, if the child had not been immunized against influenza previously), even when comparing extremes of family annual income reported by participants. The odds ratio (OR) for annual incomes >$80,000 was 1.61 (95% confidence interval [CI] 0.76 to 3.14), whereas for incomes <$40,000, the OR was 1.30 (95% CI 0.60 to 2.91). Neither of these effect estimates reached statistical significance.

**Figure 3:**
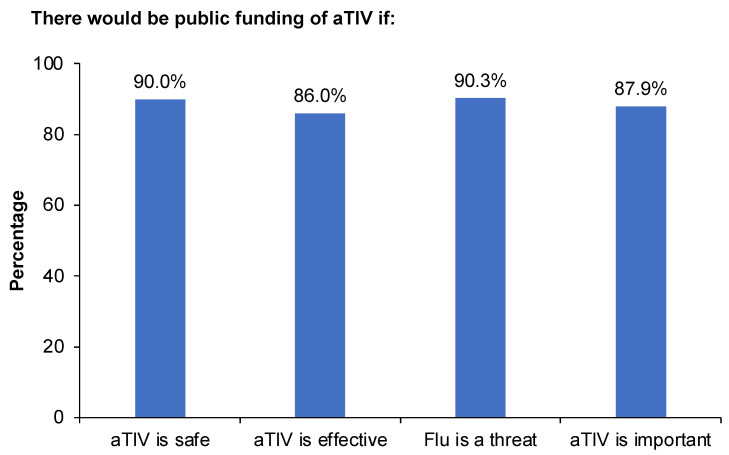
Parental beliefs about aTIV as a function of publicly funded and not publicly funded vaccine status, shown as the percentage of parents responding “strongly agree” or “moderately agree” to the following statements: “Public health would fund the adjuvanted seasonal flu vaccine and it would be cost-free to parents if”: “it was very clear that the adjuvanted seasonal flu vaccine is safe,” (aTIV is safe); “it was very clear that the adjuvanted seasonal flu vaccine is really effective” (aTIV is effective); “seasonal flu was really an important health threat to infants” (flu is a threat); “the adjuvanted seasonal flu vaccine were important for infants to have” (aTIV is important).

Although family income was not a correlate of parental acceptance of pediatric influenza vaccination with aTIV in the NPF setting, NPF status appears to send an extremely strong heuristic signal to parents concerning the safety, efficacy, and importance of this approved vaccine. Specifically, 90.0% of parents strongly agreed or agreed that “public health would fund the adjuvanted seasonal flu vaccine and it would be cost-free to parents” if it was “very clear that the adjuvanted seasonal flu vaccine is safe,” and 86.0% believed there would be public funding if the vaccine was “really effective.” A total of 90.3% of parents strongly agreed or agreed that “If seasonal flu was really an important health threat to infants,” there would be public health funding and the vaccine would be cost-free to parents, and 87.9% believed that public funding would be available if the adjuvanted seasonal flu vaccine were important for infants to have (Figure 3).

## 4. Discussion

Our findings indicate that after a brief interaction with a clinician who provides information about pediatric influenza and aTIV, most parents (>70%) intended to vaccinate their infant with aTIV if it were publicly funded. The finding that a brief and clinically feasible discussion of this nature can result in high levels of parental acceptance of infant influenza vaccination is particularly significant in the context of very low uptake rates for pediatric influenza vaccination in Canada [13,14,15]. Brief discussion of information about the range and attributes of PF and NPF influenza vaccines would appear to be key to obtaining well-informed parental consent, and it resulted in parental choice of the PF option in a plurality of cases.

An additional finding of importance involves the fact that family income was not associated with parental acceptance of a pediatric vaccine that cost $25 per dose (or $50 for two doses) or $50 (or $100 for two doses), even when contrasting NPF vaccine acceptance among parents at the highest versus the lowest levels of income assessed. Anecdotal reports of healthcare providers offering NPF vaccines only to parents who are perceived to have the means to afford such choices are not consistent with our findings.

A final set of results is both novel in the literature and of considerable importance in the setting of vaccine acceptance. With a very high degree of consensus, parents agreed that if the aTIV vaccine was really safe, really effective, really needed, and really important, “…public health would fund the vaccine and it would be cost-free to parents.” Parents appear to use public health funding as a heuristic and essential indicator of the desirability of immunizing their infants. In fact, public health funding of a vaccine involves consideration of cost, cost-effectiveness on a population basis, and opportunity costs, and is not directly informative about the safety and efficacy of an approved vaccine in connection with parental decisions about vaccinating their individual child.

Strengths of this study include collection of data from parents of infants in primary care and public health immunization settings, assessment of intentions to vaccinate infants against influenza in the PF and NPF contexts, and the implications of the current findings for understanding vaccine acceptance. A primary limitation of this research involves assessment of intentions to vaccinate with aTIV as opposed to assessment of vaccination behavior per se, because the vaccine was not yet available when this study was conducted. Although intentions are among the strongest empirical predictor of behavior [16,17], and there is value in gathering information about vaccine acceptance prior to its availability, research is needed to evaluate the specific predictive strength of such intentions. We would add that in a companion publication (Fisher et al., in this issue), we have demonstrated that a similar, brief, feasible clinician–parent discussion resulted in distinctively elevated levels of actual influenza vaccination and choice of aTIV.

## 5. Conclusions

This study demonstrated that brief HCP communication with parents about pediatric influenza vaccination is clinically feasible and is associated with high levels of parental acceptance of vaccination. Provision of information about the meaning of approved PF and NPF influenza vaccine options may be an important component of obtaining informed parental decision making. Regardless of family income, willingness of parents to vaccinate their children with aTIV decreased when the vaccine was not publicly funded. Furthermore, public funding for aTIV had a substantial impact on whether parents perceived aTIV immunization to be safe, effective, necessary, and important for their infants. Clinicians should help parents understand that NPF status does not discount the safety or efficacy of an approved vaccine in terms of disease prevention and risk reduction and should do so with all parents regardless of income.

## Figures and Tables

**Table 1:** Sociodemographic characteristics.

Characteristic	Survey Population (N = 207)
**Mean age (range)**	
Parents (years)	33 (17–54)
Children (months)	13.5 (6–24)
**Female sex, n (%)**	
Parents, n (%)	172 (83.1)
Children, n (%)	101 (48.8)
**Highest educational level attained by parent, n (%)**	
University (bachelor’s degree or higher)	106 (51.2)
Community college, technical college, or trade school	64 (30.9)
High school or equivalent	35 (16.9)
Primary school	2 (1.0)
**Parental race and ethnicity** **, n (%)**	
White	133 (64.3)
Asian	46 (22.2)
Native American	5 (2.4)
Black	6 (2.9)
Other	17 (8.2)
